# Prolongation of Anæsthesia by Chloroform

**Published:** 1864-06

**Authors:** 


Prolongation of Anæsthesia by Chloroform.—Drs. Ehrlenmeyer and Nussbaum state that by the subcutaneous injection of a solution (one-eighth grain) of morphia, the anæsthesia produced by chloroform may be prolonged without danger over many hours.

